# CO_2 _assimilation, ribulose-1,5-bisphosphate carboxylase/oxygenase, carbohydrates and photosynthetic electron transport probed by the JIP-test, of tea leaves in response to phosphorus supply

**DOI:** 10.1186/1471-2229-9-43

**Published:** 2009-04-21

**Authors:** Zheng-He Lin, Li-Song Chen, Rong-Bing Chen, Fang-Zhou Zhang, Huan-Xin Jiang, Ning Tang

**Affiliations:** 1 Institute of Horticultural Plant Physiology, Biochemistry and Molecular Biology, Fujian Agriculture and Forestry University, Fuzhou, 350002, PR China; 2 College of Horticulture, Fujian Agriculture and Forestry University, Fuzhou, 350002, PR China; 3 Tea Research Institute, Fujian Academy of Agricultural Sciences, Fuan, 355015, PR China; 4 Fujian Key Laboratory for Plant Molecular and Cell Biology, Fujian Agriculture and Forestry University, Fuzhou, 350002, PR China

## Abstract

**Background:**

Although the effects of P deficiency on tea (*Camellia sinensis *(L.) O. Kuntze) growth, P uptake and utilization as well as leaf gas exchange and Chl a fluorescence have been investigated, very little is known about the effects of P deficiency on photosynthetic electron transport, photosynthetic enzymes and carbohydrates of tea leaves. In this study, own-rooted 10-month-old tea trees were supplied three times weekly for 17 weeks with 500 mL of nutrient solution at a P concentration of 0, 40, 80, 160, 400 or 1000 μM. This objective of this study was to determine how P deficiency affects CO_2 _assimilation, Rubisco, carbohydrates and photosynthetic electron transport in tea leaves to understand the mechanism by which P deficiency leads to a decrease in CO_2 _assimilation.

**Results:**

Both root and shoot dry weight increased as P supply increased from 0 to 160 μM, then remained unchanged. P-deficient leaves from 0 to 80 μM P-treated trees showed decreased CO_2 _assimilation and stomatal conductance, but increased intercellular CO_2 _concentration. Both initial and total Rubisco activity, contents of Chl and total soluble protein in P-deficient leaves decreased to a lesser extent than CO_2 _assimilation. Contents of sucrose and starch were decreased in P-deficient leaves, whereas contents of glucose and fructose did not change significantly except for a significant increase in the lowest P leaves. OJIP transients from P-deficient leaves displayed a rise at the O-step and a depression at the P-step, accompanied by two new steps at about 150 μs (L-step) and at about 300 μs (K-step). RC/CS_o_, TR_o_/ABS (or F_v_/F_m_), ET_o_/ABS, RE_o_/ABS, maximum amplitude of IP phase, PI_abs _and PI_tot, abs _were decreased in P-deficient leaves, while V_J_, V_I _and dissipated energy were increased.

**Conclusion:**

P deficiency decreased photosynthetic electron transport capacity by impairing the whole electron transport chain from the PSII donor side up to the PSI, thus decreasing ATP content which limits RuBP regeneration, and hence, the rate of CO_2 _assimilation. Energy dissipation is enhanced to protect P-deficient leaves from photo-oxidative damage in high light.

## Background

Phosphorus (P) is one of essential macronutrients required for the normal growth and development of higher plants. Plant roots acquire P as phosphate (Pi), primarily in the form of H_2_PO_4_^-^, from the soil solution [1]. Although total Pi is abundant in many soils, the available Pi in the soil solution is commonly 1 – 2 μM due to its binding to soil mineral surfaces and fixation into organic forms [2]. Hence, P is one of the unavailable and inaccessible macronutrients in the soil [1] and is often the most limiting mineral nutrient in almost all soils [2]. Among the fertility constraints to crop production in China, low Pi availability is the primary limiting factor [3]. Pi availability is particularly limiting on the highly weathered acid soils of the tropics and subtropics, in which free iron and aluminum oxides bind native and applied Pi into forms unavailable to plants [2,3]. Therefore, Pi availability is often a major limiting factor for crop production in acid soils [2].

P deficiency affects photosynthesis in many plant species, including tea (*Camellia sinensis *(L.) O. Kuntze) [4], satsuma mandarin (*Citrus unshiu *Marc.) [5,6], pigeon pea (*Cajanus cajan *L. Millsp.) [7], soybean (*Glycine max *(L.) Merr.) [8], white clover (*Trifolium repens *L.) [9], sugar beet (*Beta vulgaris *L.) [10], tomato (*Lycopersicon esculentum *Mill.) [11], bean (*Phaseolus vulgaris *L.) [12], maize (*Zea mays *L.), sunflower (*Helianthus annuus *L.) [13]. In pigeon pea (cv. UPAS 120) [7] and tea [4], stomatal closure was at least partly responsible for the decreased photosynthetic rate under P deficiency, because the intercellular CO_2 _concentration was decreased. However, the lower CO_2 _assimilation in P-deficient leaves of soybean [14] and bean [12] was primarily caused by non-stomatal factors as the lower assimilation rate coincided with an increase of the intercellular CO_2 _concentration and the internal to ambient CO_2 _concentration ratio, respectively. Decreases in the activity and amount of Rubisco due to P deficiency have been reported for spinach (*Spinacia oleracea *L.) [15,16], sunflower [13], maize [17] and soybean [14,18]. However, experiments with sugar beet [10,19] and maize [13] showed that the effects of P deficiency on photosynthetic rate acted through RuBP regeneration rather than Rubisco activity. Jacob and Lawlor [20] concluded that the decreased CO_2 _assimilation in P-deficient sunflower and maize leaves was a consequence of a smaller ATP content and lower energy charge which limited the production of RuBP. A feedback inhibition of photosynthesis has been suggested as a cause of decreased CO_2 _assimilation at low P supply [21,22]. However, for tomato plants a decrease in starch accumulation and an increase in oxygen sensitivity of CO_2 _fixation with decreasing P supply suggest that feedback limitation is decreased under P deficiency [11,23]. P deficiency may also limit photosynthetic rate by altering leaf Chl and protein contents [24,25]. However, the decreased photosynthetic rate under P deficiency was not accompanied by decreased contents of Chl and protein per unit leaf area [10,15].

All oxygenic photosynthetic materials investigated so far using direct, time-resolved fluorescence measurement show the polyphasic rise with the basic steps of O-J-I-P [26-28]. The OJIP transient has been found to be a sensitive indicator of photosynthetic electron transport processes [29]. The kinetics of the OJIP are considered to be determined by changes in the redox state of Q_A _[28,30], but at the same time, the OJIP transient reflects the reduction of the photosynthetic electron transport chain [31]. The OJ phase represents the reduction of the acceptor side of PSII [29,31]. The JI phase parallels the reduction of the PQ-pool [29,32] and the IP phase represents the fractional reduction of the acceptor side of PSI or the last step in the reduction of the acceptor side of PSII and the amplitude of the IP phase may be a rough indicator of PSI content [31,33]. Reports concerning the effects of P deficiency on photosynthetic electron transport activity are some conflicting. Abadia et al. [34] reported that low P had no major effect on the structure and function of the photosynthetic electron transport system or on photosynthetic quantum yield of sugar beet leaves. Jacob and Lawor [20] concluded that *in vivo *photosynthetic electron transport did not limit photosynthetic capacity in P-deficient sunflower and maize leaves. However, P-deficient citrus exhibited a 6% decrease in F_v_/F_m _and a 49.5% decrease in electron transport rate [5]. Recently, Ripley et al. [35] reported that P deficiency decreased TR_o_/ABS (F_v_/F_m_), ET_o_/ABS of sorghum (*Sorghum bicolor *(L.) Moench) leaves, but had no significant effect on electron transport flux per RC (ET_o_/RC). Thus, it is not well known how P deficiency affects photosynthetic electron transport in plants.

Tea is an evergreen shrub native to China and is cultivated in humid and sub-humid of tropical, sub-tropical, and temperate regions of the world mainly on acid soils [4]. P deficiency is frequently observed in tea plantations [36,37]. For this reason, P fertilizers are being used annually in tea plantations in order to raise tea productivity and improve tea quality [4]. Although Salehi and Hajiboland [4] investigated the effects of P deficiency on tea growth, P uptake and utilization as well as leaf gas exchange and Chl a fluorescence, very little is known about the effects of P deficiency on photosynthetic electron transport, photosynthetic enzymes and carbohydrates of tea leaves. The objective of this study was to determine how P deficiency affects CO_2 _assimilation, Rubisco, non-structural carbohydrates and photosynthetic electron transport in tea leaves to understand the mechanism by which P deficiency leads to a decrease in CO_2 _assimilation.

## Results

### Leaf P content and plant growth characteristics

**Figure 1:**
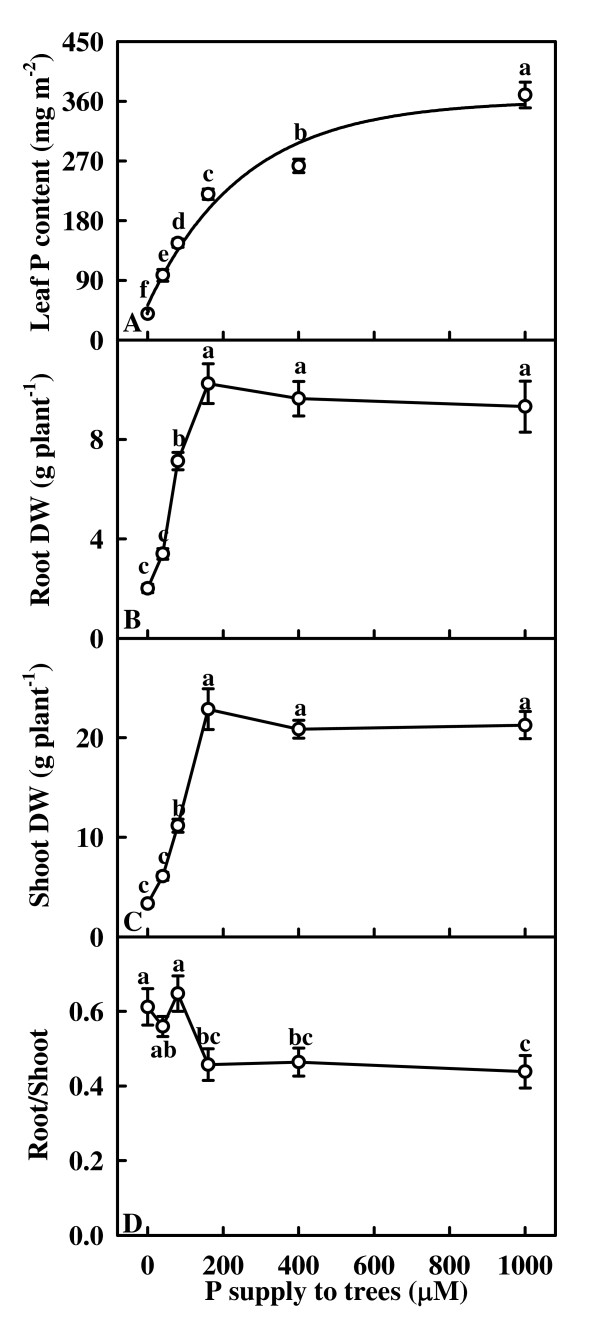
**Effects of phosphorus (P) supply on leaf P content (A), root dry weight (B), shoot dry weight (C) and root/shoot dry weight ratio (D) of tea trees**. Each point is mean ± standard error (*n* = 5 or 6). Regression equations: (A) *y *= 361.3948 – 308.8565 *e*^-0.0039*x *^(*r*^2 ^= 0.9690, *P *= 0.0055). Different letters above or below standard error bars indicate significant difference at *P *< 0.05.

As P supply decreased, leaf P content decreased curvilinearly (Fig. 1A). Both root and shoot dry weight increased as P supply increased from 0 to 160 μM, then remained unchanged (Fig. 1B and 1C). The ratio of root/shoot dry weight in the 0 to 80 μM P-treated trees was higher than in the 160 μM to 1000 μM P-treated ones (Fig. 1D).

### Specific leaf weight, Chl, Car, total soluble protein and N

**Figure 2:**
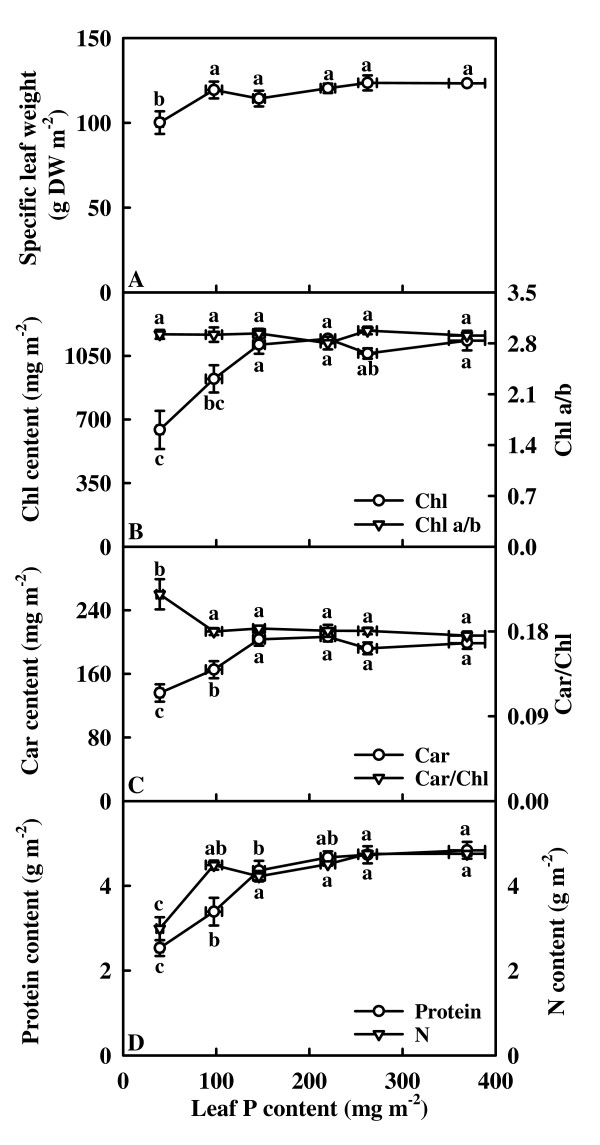
**Specific leaf weight (A), Chl content and Chl a/b ratio (B), carotenoid (Car) content and Car/Chl ratio (C), total soluble protein and N contents (D) in relation to P content in tea leaves**. Each point is mean ± standard error for the leaf P content (horizontal, *n *= 6) and the dependent variable (vertical, *n *= 5 or 6). Different letters above or below standard error bars indicate significant difference at *P *< 0.05.

Specific leaf weight did not change significantly as leaf P content decreased from 369.3 mg m^-2 ^to 97.5 mg m^-2^, then dropped significantly in the lowest P leaves (Fig. 2A). Leaf Chl (Fig. 2B), Car (Fig. 2C) and total soluble protein (Fig. 2D) contents did not change significantly as leaf P decreased from 369.3 mg m^-2 ^to 146.0 mg m^-2^, then decreased with further decreasing leaf P content. Leaf N content remained little changed with decreasing leaf P content, except for a decrease in the lowest P leaves (Fig. 2D). The ratio of Chl a/b remained unchanged over the range of leaf P content examined (Fig. 2B). The ratio of Car/Chl remained relatively constant as leaf P content decreased, except for an increase in the lowest P leaves (Fig. 2C).

### Leaf gas exchange and Rubisco

**Figure 3:**
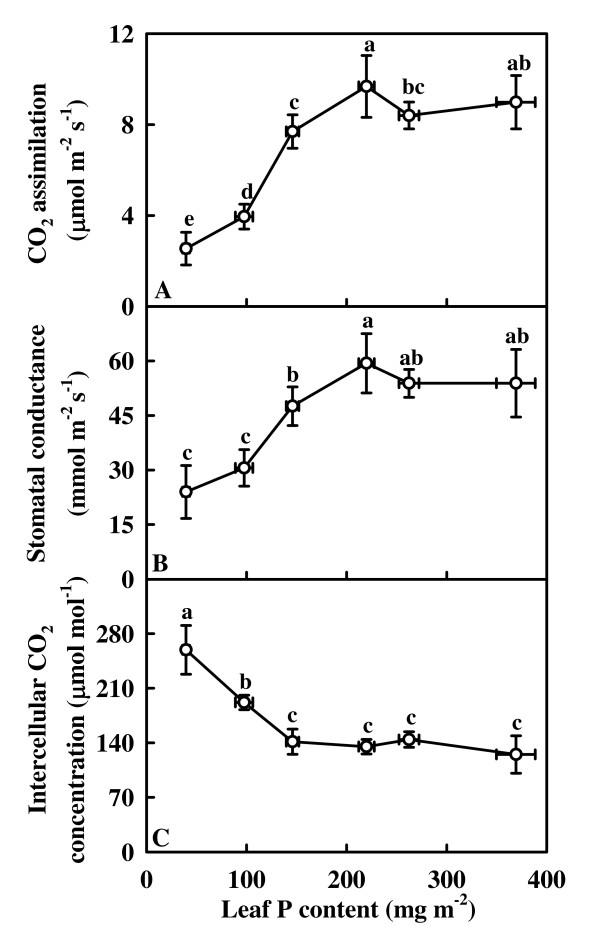
**CO_2 _assimilation (A), stomatal conductance (B), and intercellular CO_2 _concentration (C) in relation to P content in tea leaves**. Each point is mean ± standard error for the leaf P content (horizontal, *n *= 6) and the dependent variable (vertical, *n *= 5). Different letters above standard error bars indicate significant difference at *P *< 0.05.

Both CO_2 _assimilation (Fig. 3A) and stomatal conductance (Fig. 3B) increased as leaf P content increased from 39.4 mg m^-2 ^to 219.9 mg m^-2^, then remained relatively stable with further increasing leaf P content, whereas intercellular CO_2 _concentration decreased as leaf P content increased from 39.4 mg m^-2 ^to 146.0 mg m^-2^, then did not change significantly with further increasing leaf P content (Fig. 3C).

**Figure 4:**
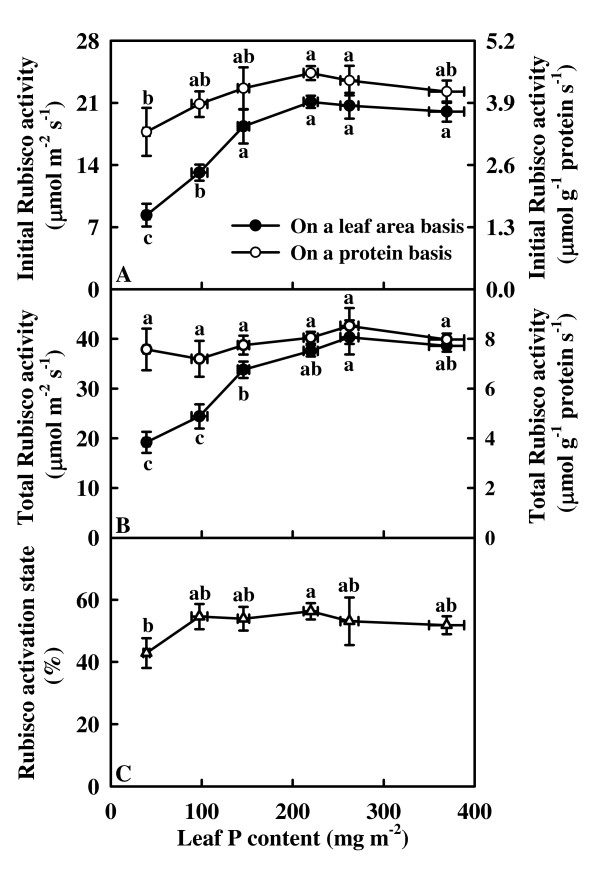
**Initial ribulose-1,5-bisphosphate carboxylase/oxygenase (Rubisco) activity (A), total Rubisco activity (B), and Rubisco activation state (C) in relation to P content in tea leaves**. Each point is mean ± standard error for the leaf P content (horizontal, *n *= 6) and the dependent variable (vertical, *n *= 5). Different letters above or below standard error bars indicate significant difference at *P *< 0.05.

On an area basis, both initial and total Rubisco activity kept relatively constant as leaf P content decreased from 369.3 mg m^-2 ^to 219.9 mg m^-2^, then decreased with further decreasing leaf P content, whereas both initial and total activity expressed on a protein basis did not change significantly over the range of leaf P content examined, except for a slight decrease in initial activity in the lowest P leaves (Fig. 4A and 4B). Rubisco activation state remained unchanged as leaf P content decreased from 369.3 mg m^-2 ^to 97.5 mg m^-2^, and then dropped in the lowest P leaves (Fig. 4C).

### Leaf nonstructural carbohydrates

**Figure 5:**
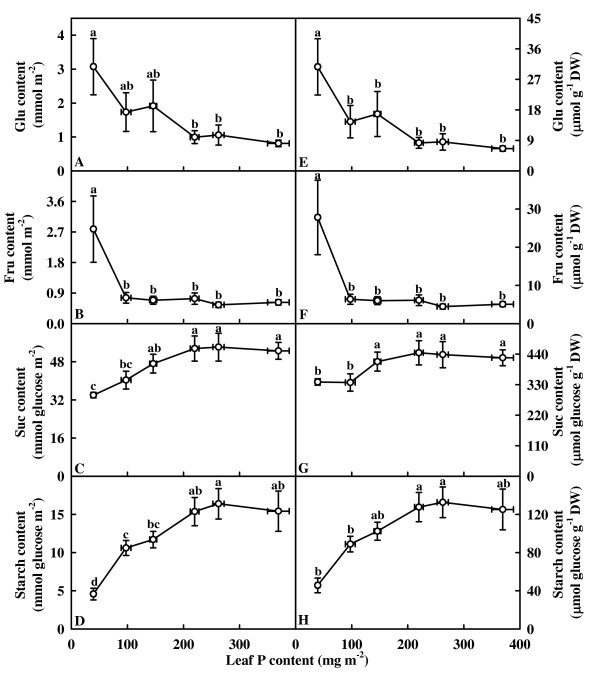
**Glucose (Glu, A and E), fructose (Fru, B and F), sucrose (Suc, C and G), and starch (D and H) contents expressed on an area (A-E) or DW (F-J) basis in relation to P content in tea leaves**. Each point is mean ± standard error for the leaf P content (horizontal, *n *= 6) and the dependent variable (vertical, *n *= 6). Different letters above standard error bars indicate significant difference at *P* < 0.05.

On an area basis, contents of glucose and fructose did not change significantly over the range of leaf P content examined except for a significant increase in the lowest P leaves (Fig. 5A and 5B). Contents of sucrose and starch remained little changed as leaf P content decreased from 369.3 mg m^-2 ^to 219.9 mg m^-2^, then decreased with further decreasing leaf P content (Fig. 5C and 5D). When expressed on a dry weight basis, sucrose content did not change significantly as leaf P content decreased from 369.3 mg m^-2 ^to 146.0 mg m^-2 ^except for a decrease in the 39.4 mg m^-2 ^and 97.5 mg m^-2 ^P leaves (Fig. 5G), whereas the other results expressed on a dry weight basis were similar to those expressed on an area basis (Fig. 5E, 5F and 5H).

### Leaf OJIP transients and related parameters

**Figure 6:**
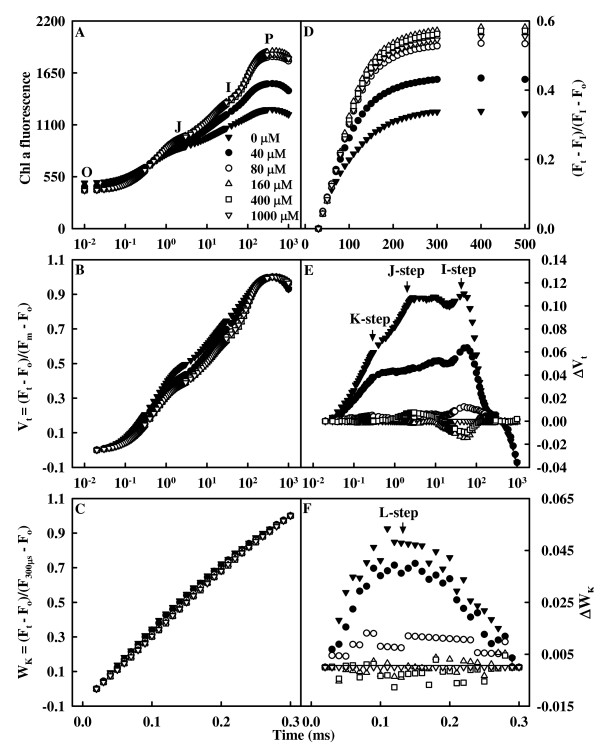
**Effects of P supply on the average Chl a fluorescence (OJIP) transients (average of 7 – 15 samples, A) and the different expressions of relative variable fluorescence: (B) between F_o _and F_m_: V_t _= (F_t _- F_o_)/(F_m _- F_o_) and (E) the differences of the six samples to the reference sample treated with 1000 μM P (ΔV_t_), (C) between F_o _and F_300 μs_: W_K _= (F_t _- F_o_)/(F_300 μs _- F_o_) and (F) the differences of the six samples to the reference sample (ΔW_K_), (D) IP phase: (F_t _- F_o_)/(F_I _- F_o_) - 1 = (F_t _- F_I_)/(F_I _- F_o_) **[71]** in dark-adapted tea leaves**.

All OJIP transients showed a typical polyphasic rise with the basic steps of O-J-I-P. OJIP transients of leaves from 0 and 40 μM P-treated trees showed a rise at the O-step and a large depression at the P-step (Fig. 6A).

Fig. 6B and 6E shows the kinetics of relative variable fluorescence at any time V_t _= (F_t _- F_o_)/(F_m _- F_o_) and the differences of normalized P-treated transients minus 1000 μM P-treated transient (ΔV_t_). The differences revealed three obvious bands: increase in the K-step (300 μs), in the 2 to 4 ms range J-step and in the 30 to 100 ms range I-step. The positive K-, J- and I-steps were very pronounced in the leaves from 0 and 40 μM P-treated trees. Fig. 6C and 6F depicts the relative variable fluorescence between F_o _and F_300 μs _(W_K_) and the differences of normalized P-treated transients minus 1000 μM P-treated transient (ΔW_K_). The differences showed a clear L-step. OJIP transients from 0 to 80 μM P-treated trees had decreased maximum amplitude of IP phase and rise time, and the end-levels were lowered by P deficiency (Fig. 6D).

**Figure 7:**
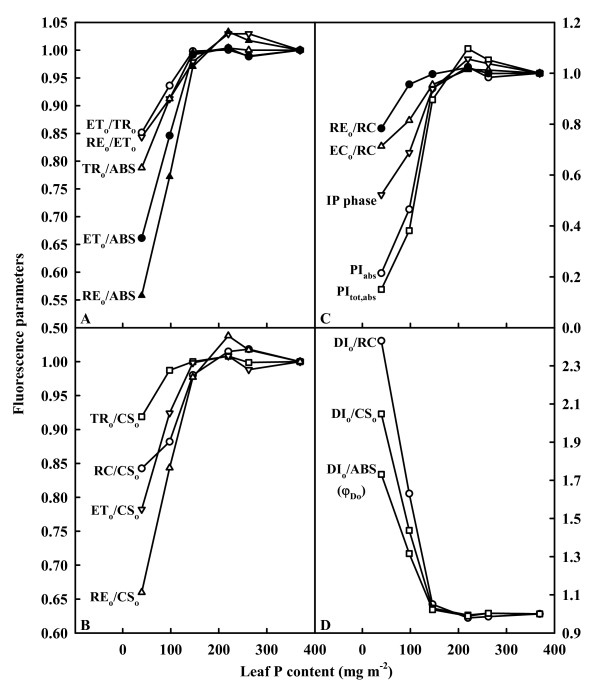
**Seventeen fluorescence parameters derived by the JIP-test from the average OJIP transients of Fig. 6A in relation to P content in tea leaves**. All the values were expressed relative to the sample treated with 1000 μM P set as 1. Maximum amplitude of IP phase = (F_m _- F_o_)/(F_I _- F_o_) - 1 [71].

Fig. 7 depicts the behavior patterns of 17 fluorescence parameters. For each parameter the values were normalized on that of the sample treated with 1000 μM P. Generally speaking, leaves from 0 to 80 μM P-treated plants had decreased ET_o_/TR_o_, RE_o_/ET_o_, TR_o_/ABS, ET_o_/ABS, RE_o_/ABS (Fig. 7A), TR_o_/CS_o_, RC/CS_o_, ET_o_/CS_o_, RE_o_/CS_o _(Fig. 7B), RE_o_/RC, EC_o_/RC, maximum amplitude of IP phase, PI_abs _and PI_tot, abs _(Fig. 7C), but increased DI_o_/RC, DI_o_/CS_o _and DI_o_/ABS (φ_Do_) (Fig. 7D).

### Leaf maximum amplitude of IP phase, PI_abs _and PI_tot, abs _in relation to CO_2 _assimilation

**Figure 8:**
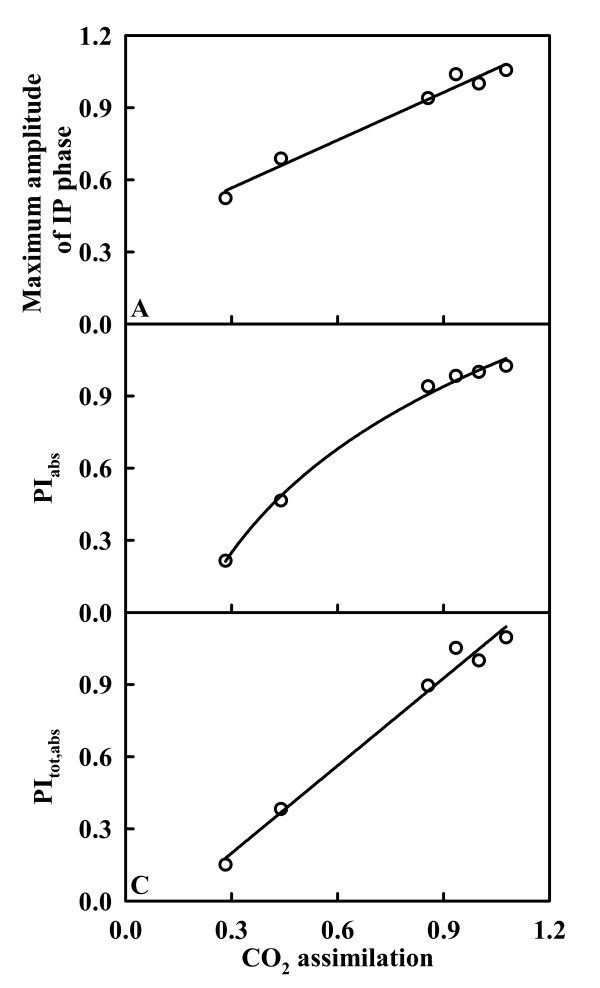
**Maximum amplitude of IP phase (A), PI_abs _(B) and PI_tot, abs _(C) in relation to CO_2 _assimilation in tea leaves**. All the values were expressed relative to the sample treated with 1000 μM P set as 1. Regression equations: (A) *y *= 0.5070 + 0.5208 × (r^2 ^= 0.9556, *P *= 0.0007); (B) *y *= -11.9070 + 12.9149 x^0.0503 ^(y^2 ^= 0.9951, *P *= 0.0003); (C) *y *= -0.1650 + 1.2127 × (y^2 ^= 0.9839, *P *< 0.0001).

Leaf CO_2 _increased linearly or curvilinearly with increasing maximum amplitude of IP phase (Fig. 8A), PI_abs _(Fig. 8B) and PI_tot, abs _(Fig. 8C), respectively.

## Discussion

Our results showed that 0, 40 and 80 μM P treatments decreased root and shoot dry weight (Fig. 1B and 1C), and foliar P content for the three treatments was lower than the sufficiency range of 1.9 to 2.5 mg g^-1 ^DW [38]. In addition, nearly all physiological and biochemical activities reached their maximum in the leaves of about 220 mg m^-2 ^from 160 μM P-treated trees (Figs. 2, 3, 4, 5, 6, 7). Based on these results, trees treated with 0, 40 or 80 μM P are considered P deficient. P deficiency resulted in an increase in the ratio of root/shoot dry weight (Fig. 1D), as previously observed in different plant species growing under different growth conditions [10,39-42]. The increase of the root/shoot dry weight ratio in response to P deficiency may be associated with stronger sink competition of the roots for P and photosynthates [7,40,43-45].

Despite decreased CO_2 _assimilation, P deficiency causes increased starch content and decreased sucrose content in leaves of several plant species including soybean [44,46], tobacco (*Nicotiana tabacum *L.) [22], spinach, barley (*Hordeum vulgare *L.) [47] and *Brachiaria *hybrid [48]. Increased partitioning of photosynthetically fixed carbon into the starch at the expense of sucrose synthesis in leaves [22,44] and decreased demand from growth [22,46,49] have been shown to contribute to increased starch accumulation in P-deficient leaves. However, a simultaneous increase in starch and sucrose contents in the leaves of P-deficient soya (*G. max *(L.) Merr.) [47], bean [50] and sugar beet [51] plants has been observed while chloroplastic and leaf levels of sugar phosphates decreased markedly [19]. In our study, P-deficient leaves had decreased sucrose (Fig. 5C and 5G) and starch (Fig. 5D and 5H) contents, as previously found for trifoliate orange (*Poncirus trifoliata *(L.) Raf.), Swingle citrumelo (*C. paradisi *Macf. × *P. trifoliata*), Carrizo citrange (*C. sinensis *(L.) Osb. × *P. trifoliata*) [52] and rice (*Oryza sativa *L.) [48]. There appears to be considerable variation in the responses of leaf carbohydrate metabolism during P deficiency. Some of the variation may result from different degree of P deficiency, time of exposure to P deficiency, plant species, light intensities used in different studies [8,22,23,47,52]. It is noteworthy that specific leaf weight decreased in the lowest P leaves (Fig. 2A). This contrasts with previous data obtained for soybean [44] and sugar beet [10], whose leaves accumulated starch under P deficiency [10,44]. Regressive analysis showed that specific leaf weight decreased linearly with decreasing leaf starch content expressed on a leaf area basis (*P *= 0.0053, data not shown). Therefore, the decrease in specific leaf weight under P deficiency may be explained, at least in part, by the decrease in starch content.

The higher intercellular CO_2 _concentration in P-deficient leaves indicates that the low CO_2 _assimilation under P deficiency (Fig. 3A and 3C) is primarily caused by non-stomatal factors, as earlier reported for soybean [14] and bean [12]. However, Salehi and Hajiboland [4] proposed that lower stomatal conductance was the main cause for the decreased CO_2 _assimilation rate in P-deficient tea leaves as the decrease in assimilation rate was accompanied by a decrease in the intercellular CO_2 _concentration. Similar result has been obtained for pigeon pea (cv. UPAS 120) [7].

It has been suggested that low sink demand limits photosynthesis under P deficiency [21,22]. In our study, however, the decrease of assimilation CO_2 _rate under P deficiency was accompanied by a decrease in the starch accumulation (Fig. 3A, 5D and 5H), as previously reported for tomato grown in high light [23]. This indicates that the production, rather that the utilization of photosynthates, is limiting. Evidence shows that soluble sugars, specifically hexoses, may repress photosynthetic gene expression, particularly of the nuclear-encoded small sub-unit of Rubisco, thus decreasing Rubisco content and CO_2 _assimilation [53]. The lack of accumulation of sucrose and hexoses in the leaves from 40 and 80 μM P-treated trees (Fig. 5A–C and 5E–G) means that the feedback repression mechanism *via *accumulation of soluble sugars does not play a major role in determining the activity of Rubisco and the rate of CO_2 _assimilation in these leaves. However, this is not to deny that the decrease in CO_2 _assimilation in the lowest P leaves can be due to the accumulation of hexoses, because the levels of glucose + fructose observed was higher than the reported threshold level (4.5 mmol m^-2^) for hexose regulation of gene expression in tobacco [54]. The decrease in initial and total Rubisco activity expressed on an area basis in response to P deficiency was probably not the primary factor limiting CO_2 _assimilation, because there was a greater decrease in CO_2 _assimilation than in Rubisco activity (Fig. 3A, 4A and 4B). In our study, the observed lower initial and total Rubisco activity expressed on an area basis in P-deficient leaves could be associated with decreased total soluble protein content (Fig. 2D), because both initial and total activity expressed on a protein basis did not change significantly over the range of leaf P content examined, except for a slight decrease in the initial activity in the lowest P leaves (Fig. 4A and 4B). The decrease in CO_2 _assimilation in P-deficient leaves cannot be attributed to a decrease in Chl and protein contents, because the decrease in leaf Chl (Fig. 2B) and total soluble protein (Fig. 2D) contents was much less than CO_2 _assimilation (Fig. 3A). Similar results have been reported for spinach [15], sugar beet [10], and bean [12].

The presence of a positive L-step at ca. 150 μs in P-deficient leaves (Fig. 6F) means that the OJIP transients from P-deficient leaves are less sigmoidal than from P-sufficient ones and that the PSII units are less grouped or less energy is being exchanged between the independent PS II units. Because the grouped conformation is more stable than the ungrouped one, the decreased grouping implies that the PSII units of P-deficient leaves have lost stability and become more fragile. Similar results have been reported for N-deficient cowpea (*Vigna unguiculata *L.) [28] and Al-treated *Citrus grandis *(L.) Osbeck [55].

The decrease of F_v_/F_m _in P-deficient leaves was caused by both a decrease in F_m _and an increase in F_o _(Fig. 6A and 7A), as previously found for tea [4], satsuma mandarin [5] and sorghum [35]. The decrease in F_v_/F_m_ under stress is considered to reflect the photoinhibitory damage to PSII complexes [56,57]. The higher F_o _may be caused by both the damage of OEC and the inactivation of some of the PSII RCs [58,59], because P-deficient leaves had decreased RC/CS_o _(Fig. 7B) and increased damage to OEC, or it may be related to the accumulation of reduced Q_A _[60], because the physiological fractional reduction of Q_A _to Q_A _^-^, as indicated by the increase in M_o _(Fig. 6B and 6E), increased in P-deficient leaves. Quenching of F_m _in P-deficient leaves may arise from the photoinhibitory quenching (qI), because an increase in F_o _with a quenched F_m _was observed in P-deficient leaves (Fig. 6A) [61] and from the xanthophyll cycle-dependent thermal energy dissipation, which was significantly higher in P-deficient satsuma mandarin leaves than in P-sufficient ones [6].

The J-step, I-step and IP phase of OJIP transients are correlated with the redox state of Q_A_, the redox state of plastoquinone, and the redox state of end acceptors at PSI electron acceptor side, respectively [27,28,30,32]. The finding that P-deficient leaves had increased V_J _and V_I _(Fig. 6B and 6E), but decreased maximum amplitude of IP phase (Fig. 6D) suggests that acceptor side of PSII became more reduced under P deficiency, but the acceptor side of PSI become more oxidized. P deficiency-induced photoinhibitory damage at PSII acceptor is also supported by the fact that F_v _(F_v _= F_m _- F_o_) was decreased in P-deficient leaves along with an increase in F_o _(Fig. 6A), which is the characteristic of photoinhibitory damage at PSII acceptor side [62]. A positive K-step appeared at ca. 300 μs in the OJIP transients in P-deficient leaves. This means that the oxygen evolving complex (OEC) is damaged [63,64]. A positive K-step has also been found in N-deficient cowpea leaves [28].

Our result showed that P deficiency decreased the total electron carriers per RC (EC_o_/RC; Fig. 7C), the yields (TR_o_/ABS (F_v_/F_m_), ET_o_/TR_o_, RE_o_/ET_o_, ET_o_/ABS, and RE_o_/ABS; Fig. 7A), the fluxes (RE_o_/RC and RE_o_/CS_o_; Fig. 7B and 7C) and the fractional reduction of the PSI end electron acceptors, as indicated by the decreased maximum amplitude of IP phase (Fig. 6D), and damaged all of the photochemical and non-photochemical redox reactions, as indicated by the decreases in PI_abs _and PI_tot, abs _(Fig. 7D). This means that leaves from P-deficient trees have a decreased capacity for electron transport, thus limiting ATP synthesis and RuBP regeneration. Lacking ATP has the consequence that Rubisco is not fully activated [65]. This might partly explain why P-deficient leaves had lower Rubisco activity and activation state (Fig. 4). Regressive analysis showed that CO_2 _assimilation decreased linearly or curvilinearly with decreasing maximum amplitude of IP phase (Fig. 8A), PI_abs _(Fig. 8B) and PI_tot, abs _(Fig. 8C), respectively. Therefore, we conclude that the decreased photosynthetic electron transport capacity, in conjunction with the lack of ATP which limit RuBP regeneration are probably the main factors contributing to decreased CO_2 _assimilation under P deficiency.

Because P-deficient leaves only utilized a small fraction of the absorbed light energy in photosynthetic electron transport, as indicated by the decreases in EC_o_/RC, ET_o_/ABS and RE_o_/ABS (Fig. 7A and 7C), compared with the P-sufficient ones, more excess excitation energy existed in P-deficient than in P-sufficient leaves in high light. Correspondingly, energy dissipation, as indicated by DI_o_/CS_o_, DI_o_/RC, and DI_o_/ABS (φ_Do_), increased in P-deficient leaves (Fig. 7D). In addition to this, the excess absorbed light in turn can lead to the production of _1_O^2 ^and reduced active oxygen species, causing damage to photosynthetic apparatus and cell structure [35,66]. Indeed, photoinhibitory damage to both donor side and acceptor side has been demonstrated to increase the production of reactive oxygen species [61,67].

## Conclusion

P deficiency decreased photosynthetic electron transport capacity by impairing the whole electron transport chain from the PSII donor side up to the PSI, thus decreasing ATP content which limits RuBP regeneration, and hence, the rate of CO_2 _assimilation. In addition to decrease light absorption by lowering Chl content, energy dissipation is enhanced to protect P-deficient leaves from photo-oxidative damage in high light.

## Methods

### Plant culture and P treatments

This study was conducted outdoors from March to November 2007 at Fujian Agriculture and Forestry University, Fuzhou. Own-rooted 10-mouth-old uniform tea (*Camellia sinensis *(L.) O. Kuntze cv. Huangguanyin) trees were transplanted into 6 L plastic pots containing sand. Each pot contained two trees, and was supplied twice weekly with 500 mL of 1/2 strength nutrient solution. Full-strength nutrient solution contained 1 mM (NH_4_)_2_SO_4_, 0.8 mM K_2_SO_4_, 1 mM KNO_3_, 2 mM Ca(NO_3_)_2_, 1 mM NH_4_H_2_PO_4_, 0.05 mM CaCl_2_, 0.6 mM MgSO_4_, 46 μM H_3_BO_3_, 9 μM MnSO_4_, 9 μM ZnSO_4_, 2 μM CuSO_4_, 2.6 μM Na_2_MoO_4_, and 30 μM Fe-EDTA. Six weeks after transplanting, the treatment was applied for 17 weeks: until the end of the experiment, each pot was supplied three times weekly with 500 mL of nutrient solution at a P concentration of 0, 40, 80, 160, 400 or 1000 μM from NH_4_H_2_PO_4 _at pH of 5.5. N concentration was maintained at a constant by the addition of (NH_4_)_2_SO_4_. At the end of the experiment, the fully-expanded (about seven weeks old) leaves from different replicates and treatments were used for all the measurements. Leaf discs (0.61 cm^2 ^in size) were collected at noon under full sun and immediately frozen in liquid N_2_. Samples were stored at -80°C until they were used for the determination of Chl, carotenoids (Car), Rubisco, carbohydrates, and protein. Special care was taken to ensure that all samples were transferred directly from liquid N_2 _to freezer of -80°C, at no time were any samples exposed to room temperature.

### Measurements of root and shoot dry weight, and specific leaf weight

At the end of the experiment, six trees per treatment from different pots were harvested. The trees were divided into roots and shoots. The plant materials were then dried at 80°C for 48 h and the dry weight measured. Specific leaf weight was calculated as the ratio of leaf dry weight to leaf area.

### Determination of leaf Chl, Car, total soluble protein, and total P

Chl, Chl a, Chl b and Car were assayed according to Lichtenthaler [68]. Total soluble protein was determined according to Bradford [69]. Total P was determined according to Fredeen et al. [44].

### Leaf gas exchange measurements

Measurements were made with a CI-301PS portable photosynthesis system (CID, WA, USA) at ambient CO_2 _concentration with a natural photosynthetic photon flux density of 1500 ± 45 μmol m^-2 ^s^-1 ^between 10:30 and 12:00 on a clear day. During measurements, leaf temperature and ambient vapor pressure were 28.0 ± 1.0°C and 1.8 ± 0.1 kPa, respectively.

### Measurements of leaf OJIP transients

OJIP transient was measured by a Handy Plant Efficiency Analyser (Handy PEA, Hansatech Instruments Limited, Norfolk, UK) according to Strasser et al. [26]. The transient was induced by red light of about 3,400 μmol m^-2 ^s^-1 ^provided by an array of three light-emitting diodes (peak 650 nm), which focused on the leaf surface to give homogenous illumination over the exposed area of the leaf. All the measurements were done with 3 h dark-adapted plants at room temperature.

### JIP test

OJIP transient was analyzed according to the JIP test. From OJIP transient, the extracted parameters (F_m_, F_20 μs_, F_50 μs_, F_100 μs_, F_300 μs_, F_J_, F_I _etc.) led to the calculation and derivation of a range of new parameters according to pervious authors [27,28,55,70,71] (see Table 1).

**Table 1:** Summary of parameters, formulae and their description using data extracted from chlorophyll a fluorescence (OJIP) transient.

Fluorescence parameters	Description
Fluorescence parameters	Description
F_t_	Fluorescence intensity at time t after onset of actinic illumination
F_50 μs_or F_20 μs_	Minimum reliable recorded fluorescence at 50 μs with the PEA- or 20 μs with Handy-PEA-fluorimeter
F_100 μs _and F_300 μs_	Fluorescence intensity at 100 and 300 μs, respectively
F_J _and F_I_	Fluorescence intensity at the J-step (2 ms) and the I-step (30 ms), respectively
F_P _(= F_m_)	Maximum recorded (= maximum possible) fluorescence at P-step
Area	Total complementary area between fluorescence induction curve and F = F_m_
Derived parameters	
Selected OJIP parameters	
F_0 _≅ F_50 μs_or F_0 _≅ F_20 μs_	Minimum fluorescence, when all PSII RCs are open
F_m _= F_P_	Maximum fluorescence, when all PSII RCs are closed
V_J _= (F_2 ms _- F_o_)/(F_m _- F_o_)	Relative variable fluorescence at the J-step (2 ms)
V_I _= (F_30 ms _- F_o_)/(F_m _- F_o_)	Relative variable fluorescence at the I-step (30 ms)
M_o _= 4 (F_300 μs _- F_o_)/(F_m _- F_o_)	Approximated initial slope (in ms^-1^) of the fluorescence transient V = f(t)
S_m _= EC_o_/RC = Area/(F_m _- F_o_)	Normalized total complementary area above the OJIP (reflecting multiple-turnover Q_A _reduction events) or total electron carriers per RC
Yields or flux ratios	
φ_Po _= TR_o_/ABS = 1-(F_o_/F_m_) = F_v_/F_m_	Maximum quantum yield of primary photochemistry at t = 0
φ_Eo _= ET_o_/ABS = (F_v_/F_m_) × (1 - V_J_)	Quantum yield for electron transport at t = 0
ψ_Eo _= ET_o_/TR_o _= 1-V_J_	Probability (at time 0) that a trapped exciton moves an electron into the electron transport chain beyond Q_A_^-^
φ_Do _= DI_o_/ABS = 1-φ_Po _= F_o_/F_m_	Quantum yield at t = 0 for energy dissipation
δ_Ro _= RE_o_/ET_o _= (1 - V_I_)/( - V_J_)	Efficiency with which an electron can move from the reduced intersystem electron acceptors to the PSI end electron acceptors
φ_Ro _= RE_o_/ABS = φ_Po _× ψ_Eo_× δ_Ro _φ	Quantum yield for the reduction of end acceptors of PSI per photon absorbed
Specific fluxes or activities expressed per reaction center (RC)	
ET_o_/RC = (M_o_/V_J_) × ψ_Eo _= (M_o_/V_J_) × (1-V_J_)	Electron transport flux per RC at t = 0
DI_o_/RC = (ABS/RC) - (TR_o_/RC)	Dissipated energy flux per RC at t = 0
RE_o_/RC = (RE_o_/ET_o_) × (ET_o_/RC)	Reduction of end acceptors at PSI electron acceptor side per RC at t = 0
	
ET_o_/CS_o _= (ABS/CS_o_) × φ_Eo_	Electron transport flux per CS at t = 0
TR_o_/CS_o _= (ABS/CS_o_) × φ_Po_	Trapped energy flux per CS at t = 0
DI_o_/CS_o _= (ABS/CS_o_) - (TR_o_/CS_o_)	Dissipated energy flux per CS at t = 0
RE_o_/CS_o _= (RE_o_/ET_o_) × (ET_o_/CS_o_)	Reduction of end acceptors at PSI electron acceptor side per CS at t = 0
Density of RCs	
RC/CS_o _=φ_Po _× (ABS/CS_o_) × (V_J_/M_o_)	Amount of active PSII RCs per CS at t = 0
Performance index	
PI_abs _= (RC/ABS) × (φ_Po_/(1 - φ_Po_)) × (ψ_o_/(1 - ψ_o_))	Performance index (PI) on absorption basis
PI_tot, abs _= (RC/ABS) × (φ_Po_/(1-φ_Po_)) × (ψ_Eo_/(1 - ψ_Eo_)) × (δ_Ro_/(1 - δ_Ro_))	Total PI, measuring the performance up to the PSI end electron acceptors

### Leaf Rubisco activity measurements

Rubisco was extracted according to Chen et al. [72]. Rubisco activity was assayed according to Cheng and Fuchigami [73] with some modifications. For initial activity, 50 μL of sample extract was added to a cuvette containing 900 μL of an assay solution, immediately followed by adding 50 μL of 10 mM RuBP, then mixing well. The change of absorbance at 340 nm was monitored for 40 s. For total activity, 50 μL of 10 mM RuBP was added 15 min later, after 50 μL of sample extract was combined with 900 μL of an assay solution to fully activate all the Rubisco. The assay solution for both initial and total activity measurements, whose final volume was 1 mL, contained 100 mM HEPES-KOH (pH 8.0), 25 mM KHCO_3_, 20 mM MgCl_2_, 3.5 mM ATP, 5 mM phosphocretaine, 5 units NAD-glyceraldehyde-3-phosphate dehydrogenase (NAD-GAPDH, EC 1.2.1.12), 5 units 3-phosphoglyceric phospokinase (PCK, EC 2.7.2.3), 17.5 units creatine phosphokinase (EC 2.7.3.2), 0.25 mM NADH, 0.5 mM RuBP, and 50 μL sample extract. Rubisco activation state was calculated as the ratio of initial activity to total activity.

### Measurements of leaf nonstructural carbohydrates

Sucrose, fructose, glucose and starch were extracted 3 times with 80% (v/v) ethanol at 80°C and determined according to Jones et al. [74].

### Experimental design and statistical analysis

There were 20 pots trees per treatment in a completely randomized design. Experiments were performed with 5–15 replicates (one tree from different pots per replicate). Differences among treatments were separated by the least significant difference (LSD) test at *P *< 0.05 level.

## Abbreviations

Chl: chlorophyll; CS: excited cross section; ET_o_/ABS: quantum yield of electron transport at t = 0; N: nitrogen; OJIP: Chl a fluorescence; P: phosphorus; PI_abs_: performance index; PI_tot, abs_: total performance index; RC: reaction center; RC/CS_o_: amount of active PSII RCs per CS at t = 0; RE_o_/ABS: quantum yield of electron transport from Q_A_^- ^to the PSI end electron acceptors; Rubisco: ribulose-1,5-bisphosphate carboxylase/oxygenase; RuBP: ribulose-1,5-bisphosphate; TR_o_/ABS or F_v_/F_m_: maximum quantum yield of primary photochemistry at t = 0; V_I_: relative variable fluorescence at the I-step; V_J_: relative variable fluorescence at the J-step.

## Authors' contributions

ZHL performed most of the experiments and wrote the manuscript. LSC designed and directed the study and revised the manuscript. RBC helped in designing the study. FZZ helped in making nutrient solution and cultivating trees. HXJ and NT helped in measuring CO_2 _assimilation and Chl a fluorescence. All authors have read and approved the final manuscript.
